# Prediction of Prednisolone Dose Correction Using Machine Learning

**DOI:** 10.1007/s41666-023-00128-3

**Published:** 2023-02-15

**Authors:** Hiroyasu Sato, Yoshinobu Kimura, Masahiro Ohba, Yoshiaki Ara, Susumu Wakabayashi, Hiroaki Watanabe

**Affiliations:** 1 Department of Pharmacy, Obihiro Kosei General Hospital, Minami 10-chome, Nishi 14-jo, Hokkaido 080-0024 Obihiro city, Japan; 2 Department of Pharmacy, Soka Municipal Hospital, Soka, Saitama Japan; 3 Department of Pharmacy, Isehara Kyodo Hospital, Isehara, Kanagawa Japan; 4 Department of Pharmacy, National Hospital Organization Shinshu Ueda Medical Center, Ueda, Nagano Japan; 5 Department of Pharmacy, Kyorin University Hospital, Mitaka, Tokyo, Japan; 6 Department of Pharmacy, Obihiro Daiichi Hospital, Obihiro, Hokkaido Japan

**Keywords:** Prescription error, Drug safety, Machine learning, Prednisolone, Imbalanced data

## Abstract

Wrong dose, a common prescription error, can cause serious patient harm, especially in the case of high-risk drugs like oral corticosteroids. This study aims to build a machine learning model to predict dose-related prescription modifications for oral prednisolone tablets (i.e., highly imbalanced data with very few positive cases). Prescription data were obtained from the electronic medical records at a single institute. Cluster analysis classified the clinical departments into six clusters with similar patterns of prednisolone prescription. Two patterns of training datasets were created with/without preprocessing by the SMOTE method. Five ML models (SVM, KNN, GB, RF, and BRF) and logistic regression (LR) models were constructed by Python. The model was internally validated by five-fold stratified cross-validation and was validated with a 30% holdout test dataset. Eighty-two thousand five hundred fifty-three prescribing data for prednisolone tablets containing 135 dose-corrected positive cases were obtained. In the original dataset (without SMOTE), only the BRF model showed a good performance (in test dataset, ROC-AUC:0.917, recall: 0.951). In the training dataset preprocessed by SMOTE, performance was improved on all models. The highest performance models with SMOTE were SVM (in test dataset, ROC-AUC: 0.820, recall: 0.659) and BRF (ROC-AUC: 0.814, recall: 0.634). Although the prescribing data for dose-related collection are highly imbalanced, various techniques such as the following have allowed us to build high-performance prediction models: data preprocessing by SMOTE, stratified cross-validation, and BRF classifier corresponding to imbalanced data. ML is useful in complicated dose audits such as oral prednisolone.

**Supplementary Information:**

The online version contains supplementary material available at 10.1007/s41666-023-00128-3.

## Introduction

Prescription errors are a serious problem that threaten patient safety. A large variation in the frequency of prescription errors has been reported in different studies, ranging from 1.1 to 41.7% [1–5]. Major categories of prescription errors include wrong drug, wrong dose, or wrong strength [6]. While pharmacists conduct prescription audits to detect inappropriate prescriptions, human checks usually have limitations. In a clinical decision support (CDS) systems or computerized physician order entry (CPOE) system, overdose alert features have been implemented in recent years. However, most dose alert features currently implemented in CDS or CPOE are simple designs that warn only when the prescribed dose exceeds the approved upper-limit dose. With this specification, it is difficult to detect incorrect doses for drugs whose appropriate dose range varies widely depending on the disease or patient.

Oral corticosteroids are potent anti-inflammatory drugs used in the treatment of many diseases. The doses of these drugs vary greatly, depending on the disease or severity or patient population. In Japan, prednisolone tablets are one of the most commonly prescribed oral corticosteroids. These tablets may be administered daily, at a maintenance dose of ≤ 5 mg/body, in chronic diseases such as collagen disease. Conversely, in the chemotherapy of malignant lymphoma, high-dose prednisolone such as 100 mg/body is prescribed. Moreover, there is a possibility of dose adjustment within the same patient, based on his/her severity of symptoms.

To accommodate the wide variation in the required dose, oral prednisolone tablets are available in some strengths (1 mg, 2.5 mg, and 5 mg tablets in Japan). When adjusting the dose from a previous prescription, wrong input of the dose or wrong selection of strengths may occur due to the existence of multiple strengths of the drug. Prednisolone tablets, which have a very wide range of approved doses, are high-risk drugs that are prone to prescription errors [7, 8]. Even with the maximum approved dose of prednisolone tablets (e.g., 100 mg/day) set as a threshold for dose alert features currently implemented in CDS or CPOE, it is difficult to detect inappropriate doses in the clinical setting.

In recent years, research using machine learning (ML) has been actively conducted in the healthcare fields [9–31], and its usefulness has been reported for solving complex problems such as diagnostic support and prognosis prediction. The aim of this study is to develop a predictive model to determine the appropriateness of the dosage of oral prednisolone tablets using a machine learning algorithm. Real-world data in the field of drug safety, such as dose errors, are imbalance with very few positive cases. We hypothesized that ML would perform better than conventional models in detecting dose-inappropriate prescription, which is highly disproportionate data. We explore the possibility that a highly accurate prediction model using machine learning will be implemented as a prescription audit function for CDS and CPOE and that it will contribute to the prevention of dose errors of high-risk drugs.

## Related Work

Clinical data are often imbalanced, i.e., some classes have much fewer instances than others. The percentage of positive cases in clinical dataset of Table 1 is imbalanced (1.6–47.2%). Without dealing with data imbalances, the minority class is often insensitive to machine learning. This problem is serious when the minority class is the target of the prediction. Several approaches have been proposed and research into solutions to overcome the problem of imbalanced datasets [32, 33].

**Table 1:** Previous works: binary prediction with multi-ML algorithm in clinical data (excluding image and text data) in 2017–2020

Authors, publish year	Outcome (prediction target)	Data size Positive case; PC (%)	ML algorithm	Optimal algorithm
Weng et al., 2017 [10]	Cardiovascular risk	378,256 patients PC 24,970(6.6%)	RF, LR, GB, NN	NN (Sen67.5%, PPV18.4%, Spec70.7%, NPV95.7%)
Zhang et al., 2019 [11]	Response to fluid challenges in patients with acute kidney injury	6682 patients PC 2,456(36.8%)	LR, XGB	XGB (AUC0.86)
Jhee et al., 2019 [12]	Late-onset preeclampsia	11,006 pregnant women PC 474(4.3%)	LR, DT, NB, SVM, RF, SGB	SGB (AUC0.924, Acc0.973, Sen0.603, Spec0.991)
Churpek et al., 2016 [13]	The combined outcome of cardiac arrest, intensive care unit transfer, or death	269,999 patients PC 16,452(6.1%)	LR, DT, KNN, SVM, NN, RF, GB	RF (AUC0.80)
Qiu et al., 2019 [14]	The live birth chance prior to the first IVF treatment	7188 women PC 2797(38.9%)	LR, RF, XGB, SVM	XGB (AUC0.70)
Lenhard et al., 2018 [15]	Treatment response in a sample of pediatric obsessive–compulsive disorder patients who had received Internet-delivered cognitive behavior therapy	61 adolescents PC 25(41.1%)	Linear, Elastic Net, RF, SVM	Linear (Acc0.83)
Hsieh et al., 2018 [16]	Mortality of patients with unplanned extubation in intensive care units	341 patients PC 60(17.6%)	ANN, LR, RF, SVM	RF (AUC0.91)
Harrington et al., 2018 [17]	The need for surgical excision in atypical ductal hyperplasia patients found on core needle biopsies	128 lesions (124 women) PC 30(23.4%)	GB, RF, radial SVM, weighted KNN, LR, elastic net	GB (AUC0.68)
Huang et al., 2018 [18]	The current acute kidney injury risk for patients undergoing percutaneous coronary intervention	947,091 patients PC 69,826(7.4%)	XGB, LR	XGB (AUC0.725)
Davoudi et al., 2018 [19]	Postoperative delirium	51,457 patients PC (3.12%)	NB, GAM, LR, SVM, RF, XGB, NN	GAM (AUC0.86, Acc0.81, Sen0.75, Spec0.81)
Corey et al., 2018 [20]	Postoperative complication risk	99,755 invasive procedural encounters (66,370 patients) PC (16.0%)	Lasso, RF, XGB	Lasso (AUC0.836, Sen0.775, Spec0.749, PPV0.362)
Yanqiu et al., 2019 [21]	Post-stroke pneumonia	13,930 patients PC 1,012(7.23%)	LR, SVM, XGB, MLP, RETAIN	RETAIN (AUC0.928, Sen090, Spec0.85, PPV0.32, NPV0.99)
Cramer et al., 2019 [22]	The incidence of pressure ulcers in the intensive care unit	50,851 admissions PC 1690(3.3%)	LR, SVM, RF, GB, NN	LR (Precision0.09, Recall0.71)
Jeong et al., 2018 [23]	Laboratory-event-related adverse drug reaction signals	1,674 drug-laboratory pairs PC 778(46.7%)	RF, LR, SVM, NN	RF (AUC0.816, Sen0.671, Spec0.780, PPV0.727, NPV0.732, F1 0.696)
Hong et al., 2018 [24]	Emergency visits and hospital admissions during radiation and chemoradiation	8134 outpatient courses PC 878(10.8%)	GB, RF, SVM, LR	GB (AUC0.798)
Du et al., 2020 [25]	Coronary heart disease for patients with hypertension	42,676 patients PC 20,156(47.2%)	XGB, SVM, LR, DT, KNN, RF	XGB (AUC0.943, Acc0.870, F1 0.855, Sen 0.820, Spec 0.914)
Lin et al., 2019 [26]	Wait times in pediatric ophthalmology outpatient clinic	37,787 patients visits	RF, elastic net, GB, SVM	RF (AUC 0.8155)
Yang et al., 2019 [27]	Heart failure in cancer patients after cancer diagnoses	17,446 patients PC 1958(11.2%)	LR, SVM, RF, GB	GB (AUC 0.9077, Sen 0.8520, Spec 0.8138)
Brisimi et al., 2018 [28]	Chronic disease hospitalizations (heart disease and diabetes)	Heart: 45,579 patients PC 3033(6.7%) Diabetes: 33,122 patients PC 5622(17.0%)	RF, ACC, SpardeLR, 4-LRT, RBF SVM, linear SVM, CT-SLSCM, CT-LSVM	heart: RF (AUC 0.8162) diabetes: RF (AUC 0.8453)
Ibrahim et al., 2020 [29]	Sepsis in the intensive care unit	13,728 records PC 4256(31.0%)	RF, XGB, SVM	XGB (AUC 0.96, Sen 0.96, Spec 0.95)
Ye et al., 2020 [30]	Elders at higher risk for fall	265,225 old patients PC 4,361(1.6%)	RF, XGB, Lasso, KNN, SVM	XGB (AUC0.807)
Jamei et al., 2017 [31]	All-cause risk of 30-day hospital readmission	335,815 patient records PC 32,718(9.7%)	NN, RF, LR	NN (Precision 0.24, Recall 0.60, AUC 0.78)

Ichikawa et al. [34] developed screening model for hyperuricemia using the random under-sampling method on highly imbalanced data with a positive rate of 1.4%. The synthetic minority over-sampling technique (SMOTE) [35] is the best-known over-sampling technique and is often used for imbalanced medical data: prediction of all-cause mortality by Sakr et al. [36] (34,212 patients data with 11.3% positive cases), prediction of stroke by Wu et al. [37] (1131 patients data with 5.0% positive case), and prediction of the risk for severe complication after bariatric surgery by Cao et al. [38] (training dataset of 37,811 patients with 3.2% positive case).

Feature selection [39] is a method of considering an imbalanced distribution by selecting the best features. De Silva et al. [40] identified prediabetes predictors from 6346 persons data with 23.4% positive case by combining feature selection and machine learning. In this study, four feature selection algorithms were used to select 15 to 30 variables depending on severity from 156 preselected variables. Ali et al. [41] improve the diagnostic performance of Parkinson’s disease by two-dimensional data selection using voice data.

Cost-sensitive learning [42] may also be used for imbalanced learning. This concept takes into account the cost of prediction errors when training a machine learning model by assuming that the cost of misclassification of the minority sample is higher than that of the majority sample. Nakamura et al. [43] used the L2-regularized logistic regression with cost-sensitive learning and CNN models to identify readmissions within 30 days of children and reported that the F-measures for both models were similar.

## Methods

### Database and Features

The data warehouse, which stores EMR data at Obihiro Kosei General Hospital, a regional center hospital in a rural area of Hokkaido in Japan, was used.

One hundred twenty-one thousand one hundred ninety prescription orders for oral prednisolone tablets (5 mg, 2.5 mg, and 1 mg), from April 1, 2012, to March 31, 2017, were extracted. Prescription orders in which one strength was prescribed more than once were excluded, assuming that the drug is taken every other day or that the instruction requires tapering (these prescriptions are sometimes seen in Japan for the purpose of reducing side effects). Also, those in which different strengths were prescribed on different days were excluded. When different strengths were prescribed for the same number of days, their doses were combined to calculate the daily dose of prednisolone. As a result, 116,285 prednisolone tablet orders were aggregated into 89,443 observations. In addition, the prescription that the previous prescription was confirmed during the investigation period were eligible (i.e., the prescription ordered prednisolone tablets for the first time was excluded). Eighty-two thousand five hundred fifty-three observations were obtained (Online Resource 1).

For the target prescriptions, department, age, gender, daily dose, and prescription days were investigated. For previous prescriptions, prescription date, daily dose, and prescription days were investigated. These features are available through the pharmacy’s dispensing system.

### Standardization of Clinical Departments

The names of the departments included in this study varied depending on the characteristics and size of the hospital. Hence, the clustering of medical departments was done to construct a general model that could be applied to hospitals of any characteristics and size. The subject data were stratified in two dimensions: daily doses (seven categories), the number of prescription days (nine categories), and the number of cases corresponding to each cell were calculated for every medical department (Online Resource 2). A Euclidean distance-based cluster analysis (Ward method) was performed, based on a distribution table normalized so that the total number of prescriptions was one for each clinical department. Nineteen clinical departments were classified into six clusters with a similar prescription trend for prednisolone tablets.

### Outcome and Variates

Prescription modification for prednisolone doses was investigated, and 135 prescriptions with dose modification were defined as positive cases. For positive cases, the dose in the pre-revision order (first edition) was investigated. Positive cases included dose-related changes (i.e., increase or decrease in the daily dose, addition and deletion of prednisolone tablets, or changes in the strength of prednisolone tablets). Changes in prescribing days, comments, and other concomitant medications were not considered positive cases. Eighty-two thousand four hundred eighteen prescriptions without dose modification were considered negative case. There was significant imbalance in the outcome, with a positive rate of 0.16%

The following features have been adapted to build a predictive model in the pharmacy prescription audit setting: the cluster of clinical departments, current daily dose (pre-revision dose in positive case), current prescription days (pre-revision days in positive case), daily dose of the previous prescription, and prescription days of the previous prescription.

### Data Preprocessing

**Fig. 1:**
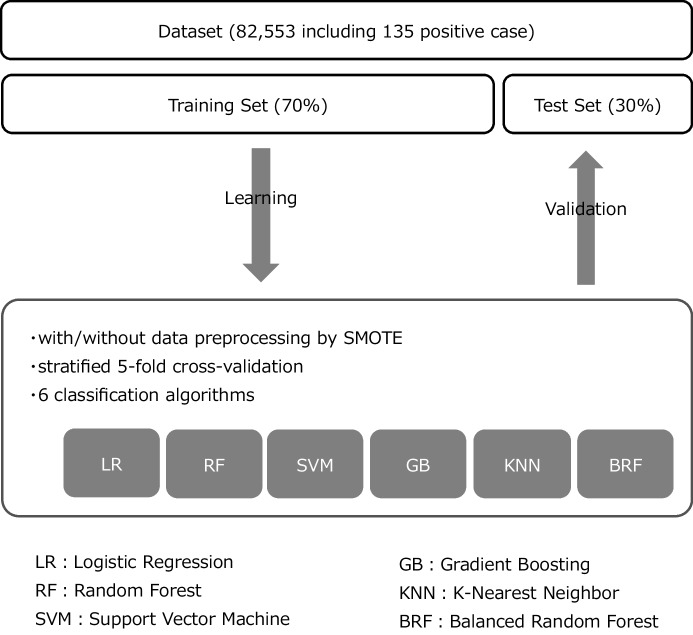
Procedure for data processing and machine learning

The data were stratified by the percentage of positive cases and divided into a 70% training dataset and a 30% test dataset (Fig. 1).

To see if dealing with imbalance data contributes to improved performance, training datasets were prepared with/without resampling preprocessing. Original training dataset includes 57,693 negative cases and 94 positive cases (positive rate: 0.16%). The preprocessed dataset was resampled by SMOTE so that the minority class was 10% of the majority (i.e., training dataset after resampling includes 57,693 negative cases and 5769 positive cases). SMOTE was performed using the Imbalanced-Learn library in the Python 3.6 language with the following settings: sampling_strategy = 0.1, k_neighbors = 5, and n_jobs = 1. The test dataset was not resampled.

### Model Development

Logistic regression (LR) and following five machine learning algorithms were used for learning by training dataset: support vector machine (SVM), k-nearest neighbor (KNN), random forest (RF), gradient boosting (GB), and balanced random forest (BRF). Then, the learning models were validated on the test dataset.

 The Python 3.6 language was used for coding the algorithm, and the Scikit-Learn library was used for all ML modules, except for BRF. The Imbalanced-Learn library was used for the latter. Hyperparameter optimization was not explored in this study.

BRF is an adaptation of random forest that under-samples the majority class, making use of the fact that random forest is an ensemble method [44]. In BRF, for each tree in the random forest, a bootstrap sample is drawn from the minority class. The same number of observations is then randomly drawn from the majority class. In the usual under-sampling method, most of the information in the majority class is lost without being used. The BRF algorithm uses the majority class data in the ensemble tree. Although studies that have used BRF for imbalanced data have been reported in various fields [45–48], very few have been reported for the healthcare field.

### Model Performance

To evaluate model performance, the following indicators were computed: area under the receiver operating characteristic curve (ROC-AUC), accuracy, precision, and recall.

1 $$\mathrm{Accuracy}=\frac{\mathrm{True}\;\mathrm{Positives}+\mathrm{True}\;\mathrm{Negatives}}{\mathrm{True}\;\mathrm{Positives}+\mathrm{False}\;\mathrm{Positives}+\mathrm{False}\;\mathrm{Negatives}+\mathrm{True}\;\mathrm{Negatives}}$$

2 $$\mathrm{Precision}=\frac{\mathrm{True}\;\mathrm{Positives}}{\mathrm{True}\;\mathrm{Positives}+\mathrm{False}\;\mathrm{Positives}}$$

3 $$\mathrm{Recall}=\frac{\mathrm{True}\;\mathrm{Positives}}{\mathrm{True}\;\mathrm{Positives}+\mathrm{False}\;\mathrm{Negatives}}$$

ROC-AUC was used as the primary performance indicator. Recall was used as the second indicator because oversight should be avoided rather than overdetermined in building a predictive model for medical safety fields.

In addition, a confusion matrix, which displays model performance via true positives, false positives, false negatives, and true negatives, was evaluated.

### Internal Validation

Internal validation was performed using k-fold stratified cross-validation (CV) for each prediction model. A normal CV divides the data into k parts in sequential order, and there may be very few or no positive cases in a sub-dataset, in imbalanced data. Stratified CV equally divides cases into positive cases and negative cases into all sub-datasets. Hence, stratified CV is used to validate imbalanced data in the healthcare field [49–53]. In this study, the data were divided evenly into five sub-datasets. The arithmetic means of the performance score, obtained from the five sub-datasets, was defined as performance after internal validation. fivefold stratified CV was only applied to training dataset.

### Statistical Analysis

All features had no missing data and no interpolation was done. For each feature in the model, positive case and negative case rates were described. Numerical information such as age, dose, days, pre-dose, and pre-days was tested by the Student’s *t* test. The chi-square test was used for sex, which is the count information. Differences in the distribution of the cluster of clinical department were compared by Fisher’s exact test. These statistical analyzes were performed using R (version 3.6.3) software.

## Results

Data related to 82,553 prednisolone prescriptions were applied to the model. Table 2 shows the background information of the subject data. Age and dose were significantly different between the groups, but their median differences were small.

**Table 2:** Characteristics of the dataset that was used in this machine learning

		Positive case (dose-correction)	Negative case (non-correction)
Data count		135	82,418
Age	Median (IQR)	61 (38.5–72)	66 (52–74)
Sex	M/F	50/85	31,081/51,472
Dose	Median (IQR)	9 (5–15)	6 (5–12.5)
Days	Median (IQR)	28 (17–35)	28 (14–49)
Pre-dose	Median (IQR)	9.5 (6–15)	7 (5–15)
Pre-days	Median (IQR)	28 (13.5–42)	28 (10–49)
Cluster of clinical department			
	CCD1	1	274
	CCD2	124	71,529
	CCD3	0	1950
	CCD4	0	17
	CCD5	1	6288
	CCD6	9	2495

IQR: first quartile–third quartile

Dose: Prednisolone dosage per a day (in positive case, pre-collection value)

Days: Administration period of prednisolone prescription (in positive case, pre-collection value)

Pre-dose: Prednisolone dosage in last prescription

Pre-days: Administration period in last prescription

CCD1: Psychiatry, plastic and reconstructive surgery, cranial nerve surgery

CCD2: General internal medicine, gastroenterology, cardiology, neurology, ophthalmology, respiratory medicine, dermatology

CCD3: Surgery, otorhinolaryngology, cardiovascular surgery, orthopedic surgery, urology

CCD4: Obstetrics and gynecology

CCD5: Hematology

CCD6: Pediatrics, radiology

The prescription patterns of prednisolone tablets differed by the clinical department (Online Resource 3). In gastroenterology, respiratory medicine, and cardiology departments, low-dose and long-term prescriptions were common. In the pediatrics department, moderate-dose and short-term prescriptions were common. In the hematology department, peaks were observed in high-dose and short-term zones because oral prednisolone was used as an anticancer drug treatment for malignant lymphomas.

**Fig. 2:**
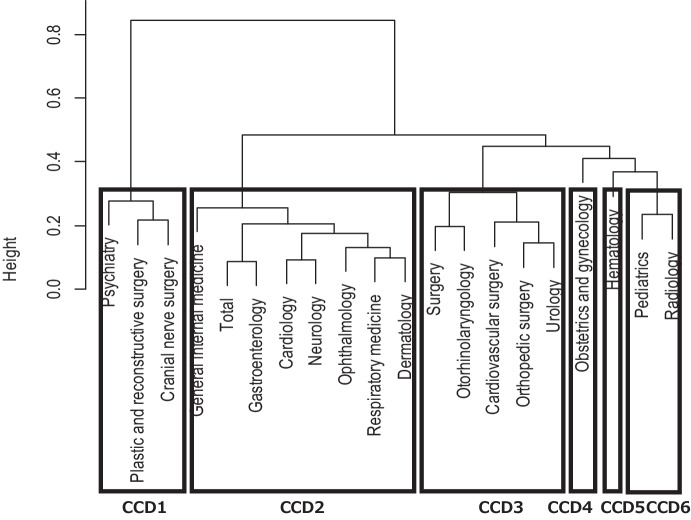
Ward clustering of clinical department with squared Euclidean distance. CCD, cluster of clinical department)

Cluster analysis classified clinical departments into six clusters with similar prescription patterns (Fig. 2). Seven departments, which had a tendency to prescribe low to moderate doses for longer duration, were grouped in one cluster, while the hematology and gynecology departments were each clustered in one clinical department. There were no positive cases in multiple clusters of clinical departments, and a significant difference was confirmed in the cluster distribution among the groups.

In the training dataset of the original data (without SMOTE), the learning results showed that the ROC-AUC of RF, GB, SVM, and BRF models was higher than that of LR (Table 3). The BRF model, which is a classifier corresponding to imbalanced data, showed the highest performance among the five ML models and had the highest recall. The LR and SVM models showed zero precision and recall and could not detect any positive cases. Due to the highly imbalanced data in this study, accuracy and ROC-AUC were high even when no positive cases were detected. Confirmation of the confusion matrix showed that no true positives existed for the LR and SVM models (Online Resource 4).

**Table 3:** Performance indicator in 5 machine learning and logistic regression models with original data (without SMOTE)

Model	Dataset	Accuracy	Precision	Recall	ROC-AUC
LR	Train	0.99837	0.00000	0.00000	0.84261
	Test	0.99834	0.00000	0.00000	0.50000
RF	Train	0.99844	0.53428	0.14912	0.89884
	Test	0.99830	0.40000	0.04878	0.52433
SVM	Train	0.99837	0.00000	0.00000	0.86501
	Test	0.99834	0.00000	0.00000	0.50000
GB	Train	0.99842	0.59500	0.18070	0.96810
	Test	0.99858	0.80000	0.19512	0.59752
KNN	Train	0.99839	0.30000	0.03216	0.69468
	Test	0.99830	0.40000	0.04878	0.52433
BRF	Train	0.88327	0.01306	0.94678	0.97346
	Test	0.88274	0.01326	0.95122	0.91692

*LR* logistic regression, *RF* random forest, *SVM* support vector machine, *GB* gradient boosting, *KNN* k-nearest neighbor, *BRF* balanced random forest

As a result of validation with the test dataset using the models learned by the original training dataset, only the BRF model showed high ROC-AUC, but the precision was very low (Table 3). The LR and SVM models were unable to classify true positives even on test dataset.

In the over-sampling data by SMOTE, performance improved for both training and test datasets for most algorithms (Table 4). Amplification of positive cases increased the proportion of true positives. The highest performing model was BRF even after resampling. With over-sampling preprocessing, precision of BRF in training dataset has also greatly improved. In the test dataset with SMOTE; there was no improvement of Recall and ROC-AUC in the BRF model, but the precision increased. In the test dataset after resampling by SMOTE, the SVM model showed the highest ROC-AUC equivalent to BRF model.

**Table 4:** Performance indicator in 5 machine learning and logistic regression models with preprocessing data by SMOTE

Model	Dataset	Accuracy	Precision	Recall	ROC-AUC
LR	Train	0.91587	0.60892	0.20870	0.86912
	Test	0.98598	0.03353	0.26829	0.62773
RF	Train	0.99784	0.99013	0.98613	0.99958
	Test	0.99830	0.47826	0.26829	0.63390
SVM	Train	0.95777	0.79727	0.71849	0.97743
	Test	0.98029	0.05389	0.65853	0.81968
GB	Train	0.99060	0.94553	0.95163	0.99868
	Test	0.99471	0.17391	0.58536	0.79037
KNN	Train	0.99180	0.92687	0.98786	0.99781
	Test	0.99228	0.09239	0.41463	0.70394
BRF	Train	0.99160	0.91850	0.99636	0.99981
	Test	0.99244	0.13131	0.63414	0.81359

*LR* logistic regression, *RF* random forest, *SVM* support vector machine, *GB* gradient boosting, *KNN* k-nearest neighbor, *BRF* balanced random forest

## Discussion

Oral corticosteroids, such as prednisolone, are associated with various adverse events including peptic ulcer, osteoporosis, hyperglycemia, and susceptibility to infections. Corticosteroids are one of the most commonly used drugs for which hospitalization due to adverse drug events is required [54]. Because corticosteroids have symptoms related to overdose and withdrawal, inappropriate dosing due to prescribing errors is a serious concern [55, 56]. Thus, it is of clinical significance to accurately detect any error related to the dose correction of prednisolone tablets. Determining the appropriate dose of oral prednisolone requires complex considerations such as calculation of daily dose (the sum of multiple standards), disease, severity, weight (especially for children), and the relationship with the previously prescribed dose. There are limits to human prescription audits, and objective support from CDS or CPOE system is desired. We developed the best ML model to detect the dose-related modified prescriptions of prednisolone tablets.

LR model without resampling, as a traditional model, could not be judged for true positives at all, and its performance was not clinically applicable. The BRF model showed highest ROC-AUC and highest recall without preprocessing. Chen et al. reported the usefulness of BRF in imbalanced data with a minority class using 2.3 to 9.7% datasets and that under-sampling appears to be superior to over-sampling [44]. The minority class of the dataset in this study was 0.16%, which was extremely imbalanced compared to the previously reported datasets. However, when combined with stratified CVs, the BRF model showed very high performance at a clinically implementable level.

By adding pre-processing of over-sampling by SMOTE, the training performance of each model was improved. While SVM performance has improved significantly, BRF has not seen much performance improvement. It seems that the BRF without resampling had already obtained sufficient performance. Another reason may be that under-sampling by BRF after over-sampling by SMOTE offset the preprocessing effect. The SVM model after SMOTE resampling (SMOTE + SVM) showed the highest ROC-AUC for the test dataset, slightly above SMOTE + BRF. Because SVM classifiers are very sensitive to imbalanced data [57], SMOTE + SVM has been reported to be a good combination [58] and was considered to be the best algorithm candidate in future developmental studies. In this study, default parameters are used for machine learning. Adjusting hyperparameters can further improve performance. Only one type of SMOTE setting, which sets the amplification factor of the minority class to 10% of that of the majority class, is being considered. It has not been verified whether this amplification setting is optimal. Since the data in this study were obtained from a single facility, the applicability of this model in other facilities needs to be further investigated.

In the prediction in the medical safety area, it is important to catch all positive cases. Therefore, in this study, recall was more important than precision. Since in this study, positive cases were defined as “dose-related prescribing correction cases,” various types of positive cases were observed. High-risk prescriptions with prednisolone dose errors that must be detected include the wrong selection of strength (a case in which instead of a dose reduction from 1 tablet of 5 mg [5 mg/day] to 4 tablets of 1 mg [4 mg/day], 4 tablets of 5 mg [20 mg/day] were prescribed), wrong selection of dose unit (a case in which instead of a prescription of 5 mg, 5 tablets were prescribed), and typing error (a case in which instead of 10 mg, 1 mg or 100 mg was prescribed). On the other hand, positive cases in this study also include cases with little risk in which the dose was adjusted after the prescription order, according to the patient’s condition (for example, a correction from 5 to 4 mg after the order). In this study, since the data were collected retrospectively, it was not possible to investigate the reasons for dose correction. Because in the case of an acute exacerbation, prednisolone may suddenly be administered at high doses; it was also difficult to objectively define “high-risk error dose,” even if the dose is many times higher than the previous prescription. In this study, assuming a secondary audit by a pharmacist, we attempted to construct a first screening model to detect any dose modification. Therefore, the precision of the best ML model in this study was expected to be low to some extent.

Because the optimal ML model constructed in this study is assumed to be used in the setting of community pharmacies, the input variables were limited to the following information described in Japanese prescriptions (i.e. the information which could be recognized by the community pharmacists): patient age, gender, clinical department, and previous prescription information. Features such as laboratory data, indications, severity, and weight were not used in this study because they are difficult to obtain at many pharmacies in Japan. The addition of these variables is expected to improve predictive performance.

Furthermore, the input variables of different departments were clustered in this study. It was observed that the prednisolone prescribing pattern was different for each clinic, and clinic information was considered to be an influential factor in erroneous dose detection. However, the name of the department name can vary, depending on the number of beds and features of the hospital. For example, in the university hospitals, medical departments are highly subdivided (e.g. “endocrinology and metabolism,” “nephrology,” “diabetes”), but in regional small or medium hospitals, integrated names are often listed (e.g. “general internal medicine”). In this study, the clustering of clinical departments was performed using prescription patterns of dose and days to construct a general-purpose model. However, since there has been no report on the standardization of the clustering of clinical departments for prednisolone prescription patterns, it is necessary to verify our method as the clustering by the Ward’s method with Euclidean and thresholds for dividing doses and days to create frequency distributions.

There are few reports of ML adaptation to medication error such as prescription audits [59–64]. To the best of our knowledge, this is the first report that ML was considered for prescription audits of drugs with a very wide range of clinical doses, such as oral steroids.

The results of this study show that even in the field of clinical drug safety, where the positive cases are few, ML dealing with imbalanced data may be useful for problems that cannot be solved by mathematical models. Prednisolone tablets, a commonly prescribed oral corticosteroid, is a high-risk drug, and its overdose or underdose is a significant risk to patients. Since this study targets prescription audit settings in pharmacies, a prediction model was constructed with an emphasis on recall to prevent under-detection. As a result, although we built a high-performance machine learning model, prescriptions that detect inappropriate doses of prednisolone should be reviewed by pharmacists for the necessary of prescription question to physicians. Accurate warning regarding prescription dosage errors with ML will be beneficial to both healthcare professionals and patients. It is expected that ML will be extensively utilized in clinical drug safety management, including prescription audits, to prevent serious incidents.

## Supplementary Information

Below is the link to the electronic supplementary material.

Supplementary file1 (PDF 28 KB)

Supplementary file2 (PDF 18 KB)

Supplementary file3 (PDF 949 KB)

Supplementary file4 (PDF 158 KB)

## Data Availability

The dataset is not open. The datasets generated and/or analyzed during the current study are available from the corresponding author on reasonable request.
