# Supplementary figures and images for: Prediction of Prednisolone Dose Correction Using Machine Learning
Source: J Healthc Inform Res. 2023 Feb 15;7(1):84–103. doi: 10.1007/s41666-023-00128-3 (PMC9995628; doi:10.1007/s41666-023-00128-3)

Online Resource 1:

Procedure for extracting data and collection of variables

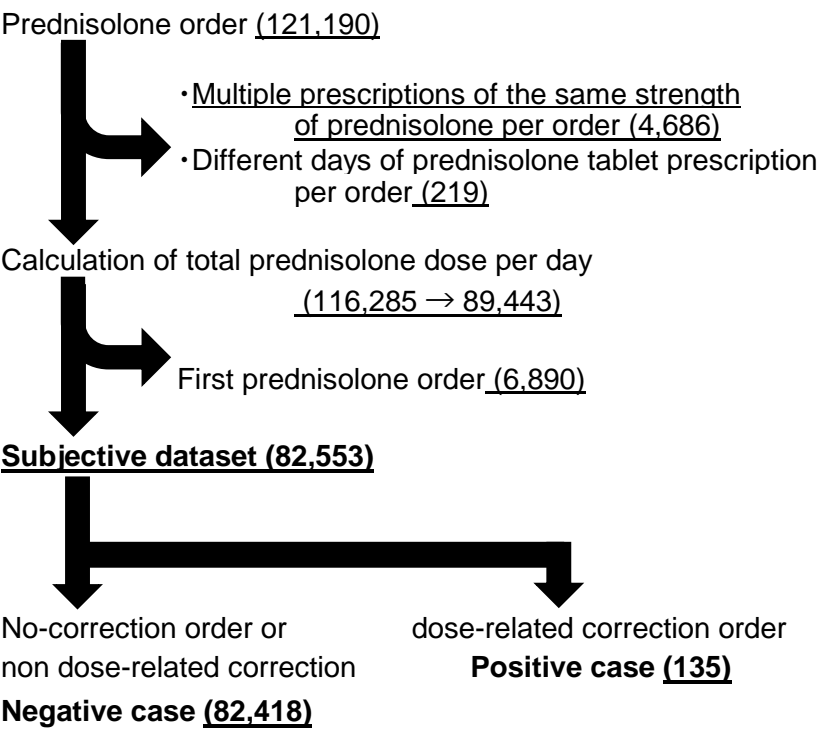

Supplement: Supplementary file 1 — Supplementary file1 (PDF 28 KB) [file 41666_2023_128_MOESM1_ESM.pdf]
