# Supplementary material for: Prediction of Prednisolone Dose Correction Using Machine Learning
Source: J Healthc Inform Res. 2023 Feb 15;7(1):84–103. doi: 10.1007/s41666-023-00128-3 (PMC9995628; doi:10.1007/s41666-023-00128-3)
Supplement: Supplementary file 2 — Supplementary file2 (PDF 18 KB) [file 41666_2023_128_MOESM2_ESM.pdf]

Prediction of prednisolone dose correction using machine learning

Journal of Healthcare Informatics Research

Hiroyasu Sato (Obihiro Kosei General Hospital, hiroyasu.sato@ja-hokkaidoukouseiren.or.jp)

Online Resource 2:

Frequency distribution table of prednisolone prescription pattern in full subject data

		Daily Dose of Prednisolone (mg)						
		0-2.5	2.6-5	5.1-10	11-20	21-30	31-75	76-
Prescription Days (day)	1-3	0.00207	0.00687	0.00734	0.01273	0.01066	0.00618	0.00009
	4-5	0.00184	0.00430	0.00635	0.01060	0.00908	0.02261	0.00315
	6-9	0.01114	0.02986	0.02423	0.02627	0.02406	0.02512	0.00016
	10-14	0.01347	0.02507	0.01850	0.02281	0.01082	0.00312	0
	15-21	0.00720	0.01593	0.00973	0.00892	0.00343	0.00048	0
	22-30	0.04258	0.10146	0.03833	0.02507	0.00362	0.00021	0
	31-60	0.06603	0.14142	0.03575	0.01277	0.00096	0.00001	0
	61-100	0.04388	0.08880	0.01126	0.00093	0.00001	0	0
	100-	0.00070	0.00139	0.00027	0	0	0	0

The number of each cell shows the ratio when the sum of all cells is 1.

This table was calculated for each clinical department in this study.
