# Supplementary material for: Prediction of Prednisolone Dose Correction Using Machine Learning
Source: J Healthc Inform Res. 2023 Feb 15;7(1):84–103. doi: 10.1007/s41666-023-00128-3 (PMC9995628; doi:10.1007/s41666-023-00128-3)

Prediction of prednisolone dose correction using machine learning
Journal of Healthcare Informatics Research
Hiroyasu Sato (Obihiro Kosei General Hospital, hiroyasu.sato@ja-hokkaidoukouseiren.or.jp)

Online Resource 3:
Prescribing patterns of prednisolone for each clinical department

Cluster of Clinical Department (CCD) 1

Surgery

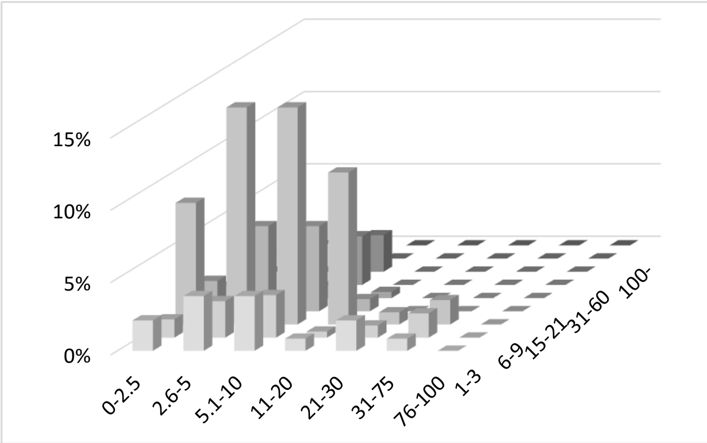

Otorhinolaryngology

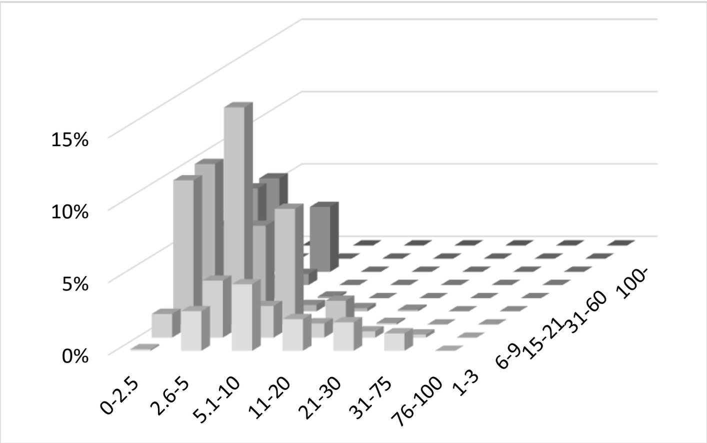

Cardiovascular surgery

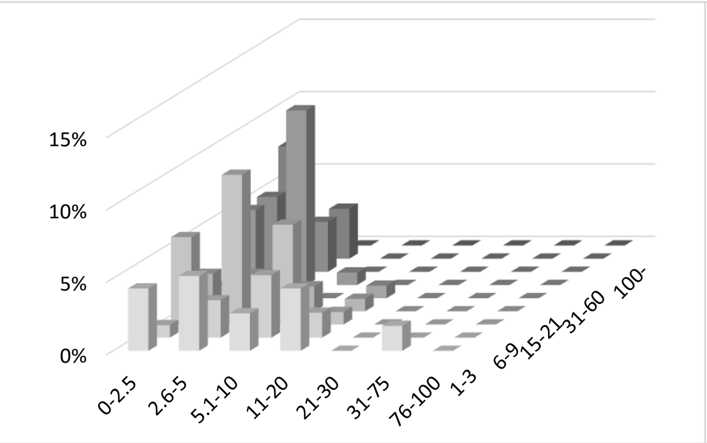

Orthopedic surgery

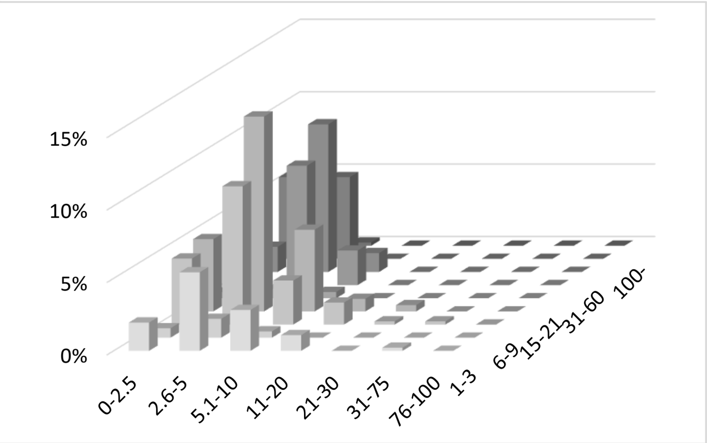

Urology

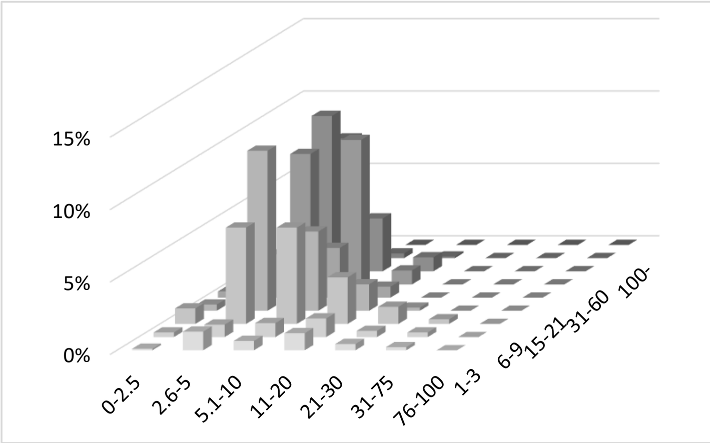

CCD2

General internal medicine

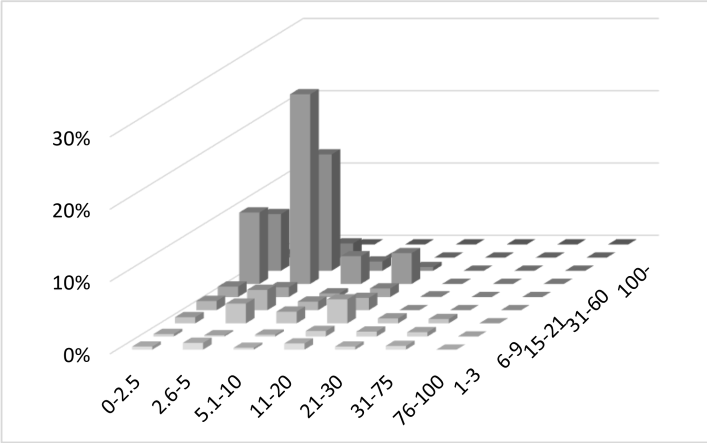

Gastroenterology

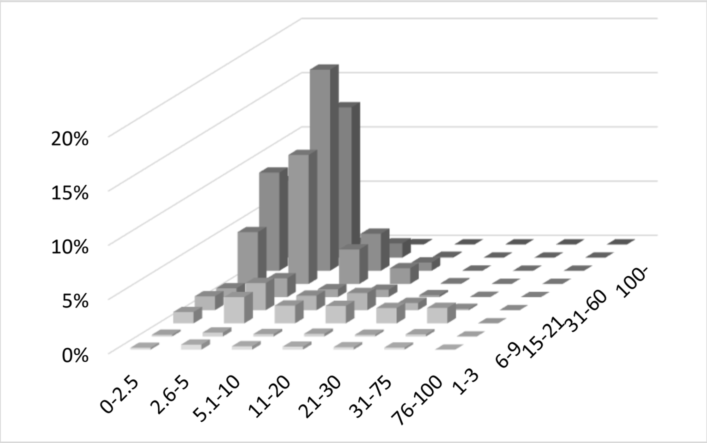

Cardiology

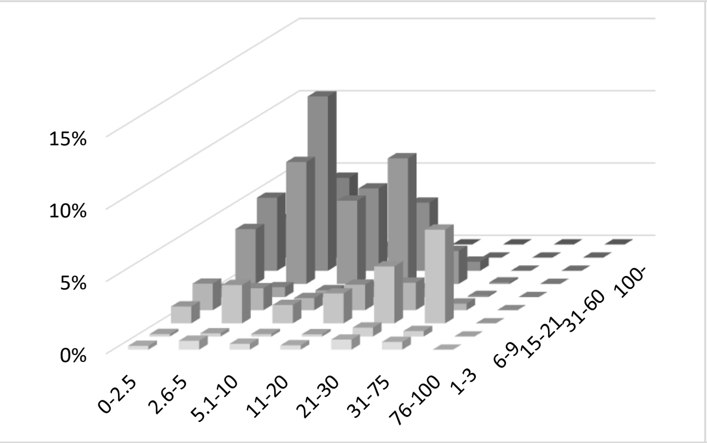

Neurology

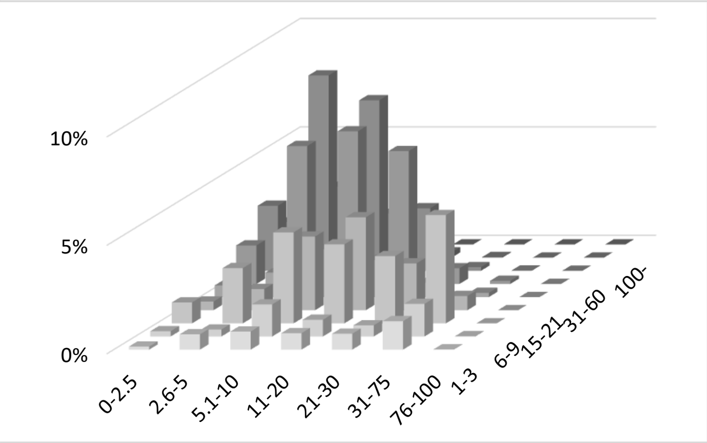

Ophthalmology

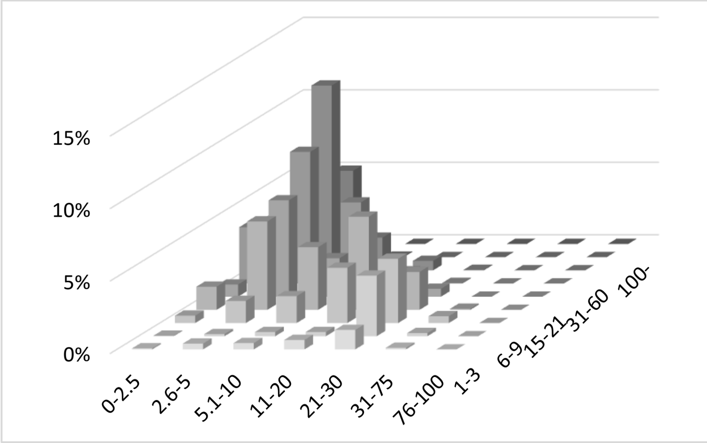

Respiratory medicine

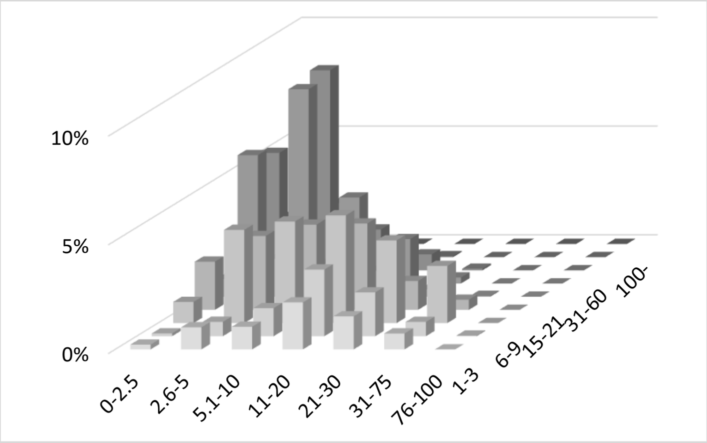

Dermatology

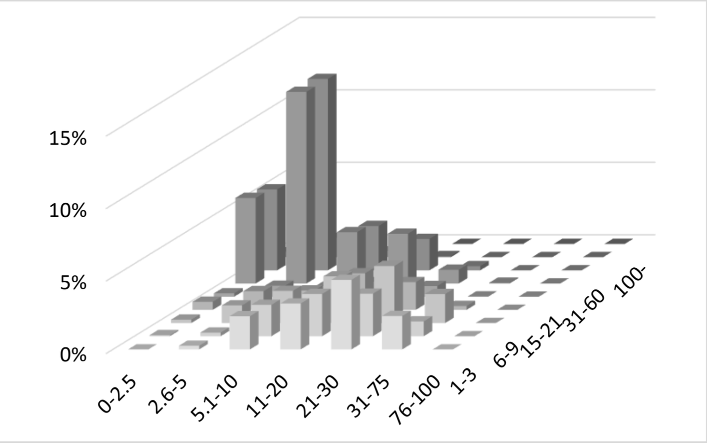

CCD3

Hematology

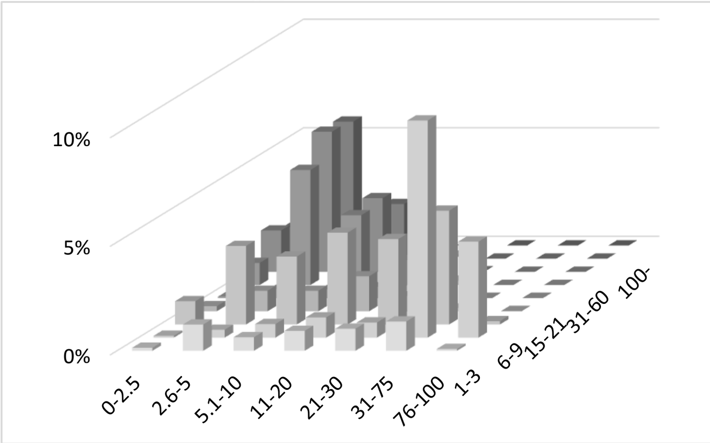

CCD4

Pediatrics

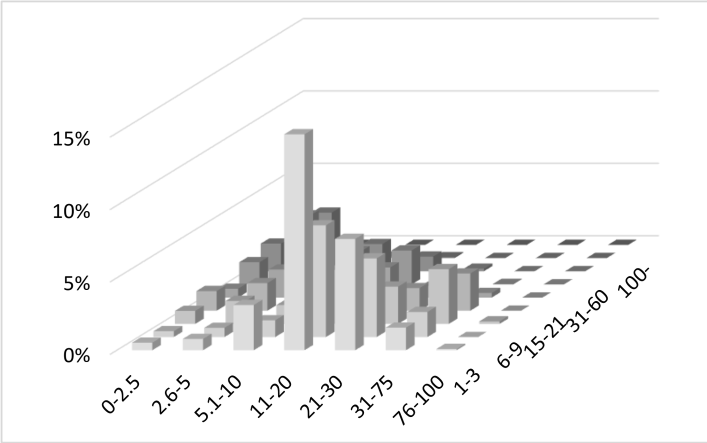

Radiology

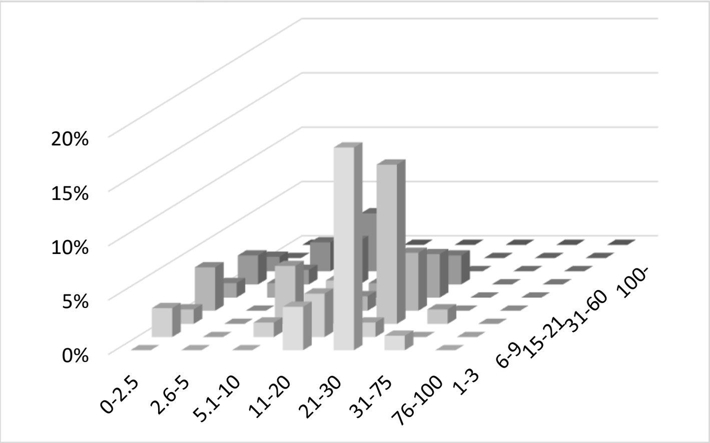

CCD5

Obstetrics and gynecology

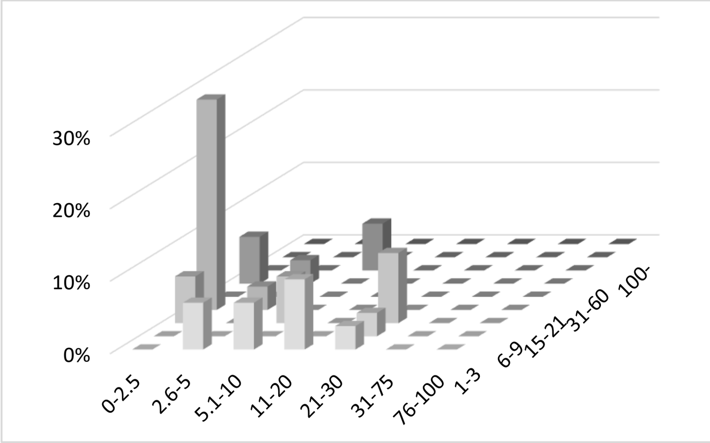

CCD6

Psychiatry

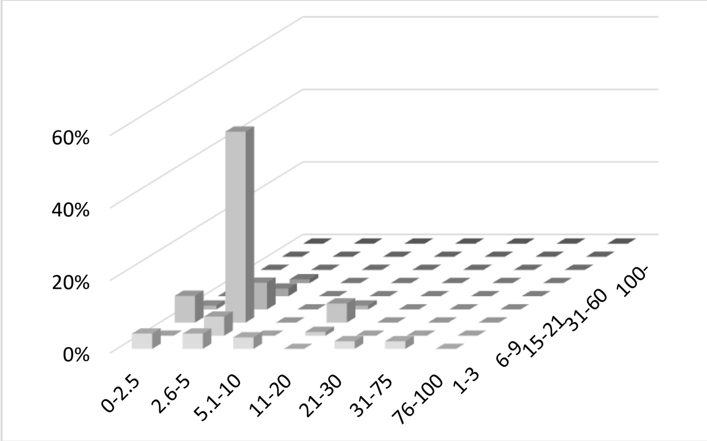

Cranial nerve surgery

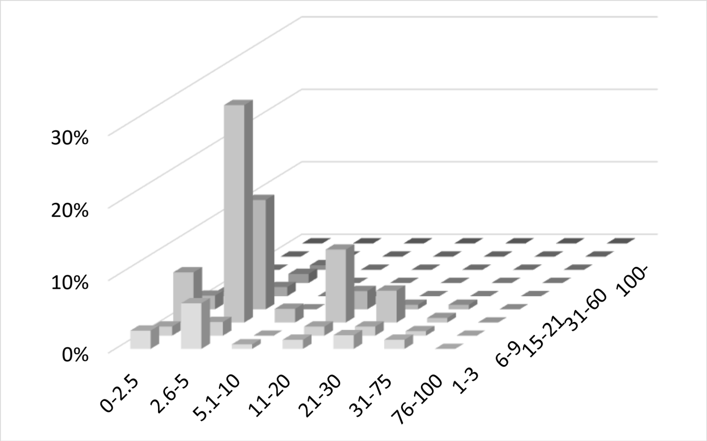

Plastic and reconstructive surgery

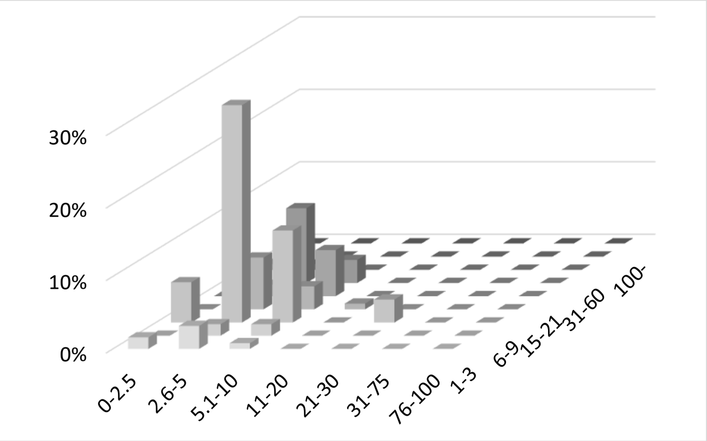

Supplement: Supplementary file 3 — Supplementary file3 (PDF 949 KB) [file 41666_2023_128_MOESM3_ESM.pdf]
