# Supplementary material for: Prediction of Prednisolone Dose Correction Using Machine Learning
Source: J Healthc Inform Res. 2023 Feb 15;7(1):84–103. doi: 10.1007/s41666-023-00128-3 (PMC9995628; doi:10.1007/s41666-023-00128-3)
Supplement: Supplementary file 4 — Supplementary file4 (PDF 158 KB) [file 41666_2023_128_MOESM4_ESM.pdf]

Hiroyasu Sato (Obihiro Kosei General Hospital, hiroyasu.sato@ja-hokkaidoukouseiren.or.jp)

Online Resource 4:
Confusion matrix in 5 machine learning and logistic regression models for training dataset of original data (without SMOTE)

	RF		LR		SVM	
	Prediction Negative	Prediction Positive	Prediction Negative	Prediction Positive	Prediction Negative	Prediction Positive
True Negative	57,683	10	57,693	0	57,693	0
True Positive	84	10	94	0	94	0

	GB		KNN		BRF	
	Prediction Negative	Prediction Positive	Prediction Negative	Prediction Positive	Prediction Negative	Prediction Positive
True Negative	57,679	14	57,691	2	50,953	6,740
True Positive	77	17	91	3	5	89

RF: Random Forest
LR: Logistic Regression
SVM: Support Vector Machine
GB: Gradient Boosting
KNN: k-Nearest Neighbor
BRF: Balanced Random Forest
